# Chemical Diversity and Potential Target Network of Woody Peony Flower Essential Oil from Eleven Representative Cultivars (*Paeonia* × *suffruticosa* Andr.)

**DOI:** 10.3390/molecules27092829

**Published:** 2022-04-29

**Authors:** Gaoming Lei, Chaoying Song, Xinyue Wen, Guoyu Gao, Yanjie Qi

**Affiliations:** Department of Pharmaceutical Sciences, School of Basic Medical Sciences, Henan University of Science and Technology, Luoyang 471023, China; 200320201100@stu.haust.edu.cn (C.S.); 191418030220@stu.haust.edu.cn (X.W.); 191418030201@stu.haust.edu.cn (G.G.); 171415020114@stu.haust.edu.cn (Y.Q.)

**Keywords:** chemical diversity, essential oil, woody peony cultivar (*Paeonia* × *suffruticosa* Andr.), potential target network, enrichment analysis, central nervous system

## Abstract

Woody peony (*Paeonia × suffruticosa* Andr.) has many cultivars with genetic variances. The flower essential oil is valued in cosmetics and fragrances. This study was to investigate the chemical diversity of essential oils of eleven representative cultivars and their potential target network. Hydro-distillation afforded yields of 0.11–0.25%. Essential oils were analyzed by GC-MS and GC-FID which identified 105 compounds. Three clusters emerged from multivariate analysis, representative of phloroglucinol trimethyl ether (‘Caihui’), citronellol (‘Jingyu’, ‘Zhaofen’ and ‘Baiyuan Zhenghui’) and mixed (the rest of the cultivars) chemotypes. ‘Zhaofen’ and ‘Jingyu’ also exhibited low levels of other rose-related compounds. The main components were subjected to a target network approach. Drug-likeness screening gave 20 compounds with predictive blood–brain barrier permeation. Compound target network identified six key compounds, namely nerol, citronellol, geraniol, geranic acid, *cis*-3-hexen-1-ol and 1-hexanol. Top enriched terms in GO, KEGG and DisGeNET were mostly related to the central nervous system (CNS). Protein—protein interactions revealed a core network of 14 targets, 11 of which were CNS-related (targets for antidepressants, analgesics, antipsychotics, anti-Alzheimer’s and anti-Parkinson’s agents). This work provides useful information on the production of woody peony essential oils with specific chemotypes and reveals their potential importance in aromatherapy for alternative treatment of CNS disorders.

## 1. Introduction

Woody peony is a shrub in section Moutan DC., genus *Paeonia* L., family Paeoniaceae (synonymously subfamily Paeonioideae in family Ranunculaceae). The cultivated species of the woody peony mainly refers to *Paeonia* × *suffruticosa* Andr. The plant height ranges from 0.5 to 2 m. It has alternate leaves, with a mostly bi-ternate compound leaf. The flower is solitary, terminal and double. The petal is obovate with various colors such as white, pink, red, purplish red, purple, dark purple or green. The flowering period is from April to May, and the fruiting period is from August to September. This species has a long history of cultivation in China and is planted countrywide, especially in the Central Plains region. The number of cultivars is up to hundreds of, or nearly one thousand, cultivars. Its flower is known as the national flower of China. It is also cultivated worldwide, especially in Japan, France, Britain and America [1,2].

The flower of the woody peony has long been used in traditional Chinese medicine, mainly for the treatment of irregular menstruation and menstrual abdominal pain [3]. The essential oil of the woody peony flower is especially valuable, which is used in cosmetics and fragrances. Zhang et al. [4] analyzed *P.* × *suffruticosa* petal essential oil obtained by microwave-assisted steam distillation, and found it contained tetracosane, hexacosane and docosane, and low levels of α-terpineol, geraniol and linalool. In a study using GC × GC coupled with TOF-MS, the petal essential oils of two woody peony cultivars were characterized by alcohols, aldehydes, alkanes, terpenes and acids [5]. Han and Bhat [6] found that *P.* × *suffruticosa* flower bud essential oil contained high levels of alkanes and low levels of alcohols, ketones and benzenoids, which showed an antimicrobial effect against food-borne bacteria. In another study, supercritical extraction from the petals of eleven *P.* × *suffruticosa* cultivars afforded (*Z*)-5-dodecenyl acetate, nonadecane, ethyl linoleate, (*Z*)-5-nonadecene, heneicosane and linalool oxide as the main components with varied contents. Moreover, the essential oils of some cultivars showed antioxidant activities [7]. Our previous studies mainly focused on the flower hydrolate extracts of some *P.* × *suffruticosa* cultivars, which revealed the predominance of benzene ethanol, phloroglucinol trimethyl ether, citronellol + geraniol or a balance of these types of compounds in different cultivars [8,9].

The chemical diversity of plant essential oils depends on geographical or ecological factors [10], genotypes [11,12], harvest, post-harvest and extraction methods [13,14]. The genotype is the major factor that determines the chemical type. The chemical type further determines specific pharmacological effects and aromatherapy applications. Active essential oil compounds work through interactions with related targets in the human body. Moreover, there might be a network of targets for the complex mixture of compounds such as essential oils [15,16,17].

Up to now, studies on the chemical diversity of woody peony flower essential oils, especially those utilizing multivariate analyses, are rather limited. Given that *P. × suffruticosa* has many cultivars with genetic variances, relevant studies are needed. Moreover, there are no reports on the potential target network of the woody peony flower essential oils. The network study of target proteins is important for giving insight into the in vivo mechanisms of essential oils. Therefore, the objectives of this work were to investigate the chemical diversity of the flower essential oils of eleven representative woody peony cultivars, and to further reveal the potential target network of the essential oil components.

## 2. Results

### 2.1. Hydro-Distillation Yields of the Essential Oils of Woody Peony Flowers

**Figure 1:**
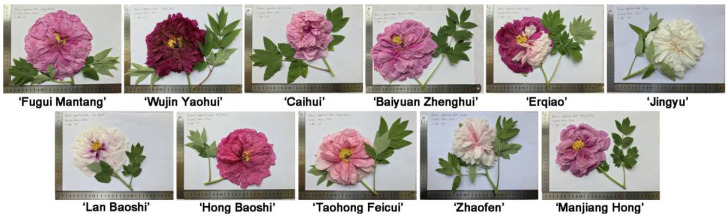
Photographs of flowers of the eleven woody peony cultivars (*Paeonia × suffruticosa* Andr.).

The flowers of the eleven woody peony cultivars are shown in Figure 1. The flower colors varied considerably. Notably, the ‘Jingyu’ flowers presented white, and ‘Wujin Yaohui’ presented dark purplish red. ‘Erqiao’ presented purplish red and pink in one flower, or in some other cases, in one plant.

Hydro-distillation of the fresh flowers afforded essential oil yields of 0.11–0.25% (*w*/*w*, on dry basis) from the eleven cultivars (Table 1). Results of analysis of variance (ANOVA) showed that the essential oil yield was influenced by the factor of woody peony cultivar (*p* < 0.05). The post-hoc test which utilized Tukey’s method for multiple comparisons suggested that the yield values basically fell into two groups overlapping partially. The yield from ‘Taohong Feicui’ belonging to group A was relatively high, whereas those from ‘Jingyu’, ‘Wujin Yaohui’, ‘Zhaofen’, and ‘Fugui Mantang’ belonging to group B was relatively low.

### 2.2. Comparative Chemical Composition of the Flower Essential Oils

**Figure 2:**
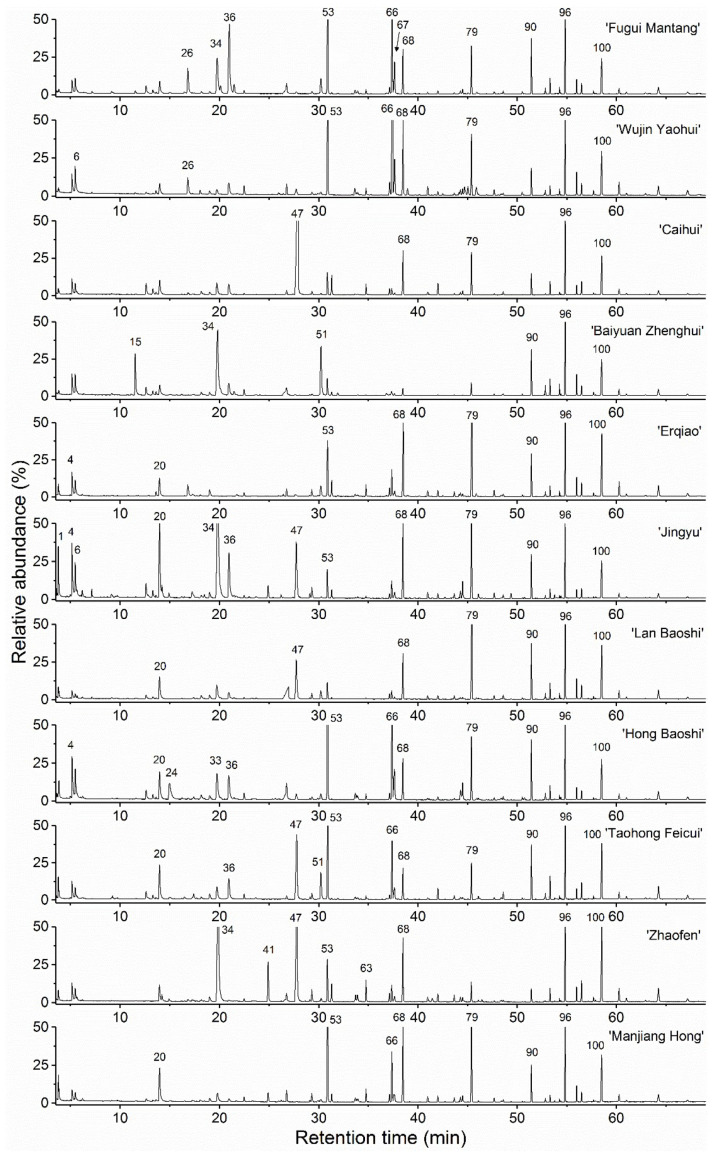
Typical gas chromatograms of flower essential oils of the eleven woody peony cultivars (*Paeonia × suffruticosa* Andr.). The main chromatographic peaks are noted with numbers. The numbering of essential oil components is specified in Table 2. Their identification was based on mass spectra and retention indices.

**Figure 3:**
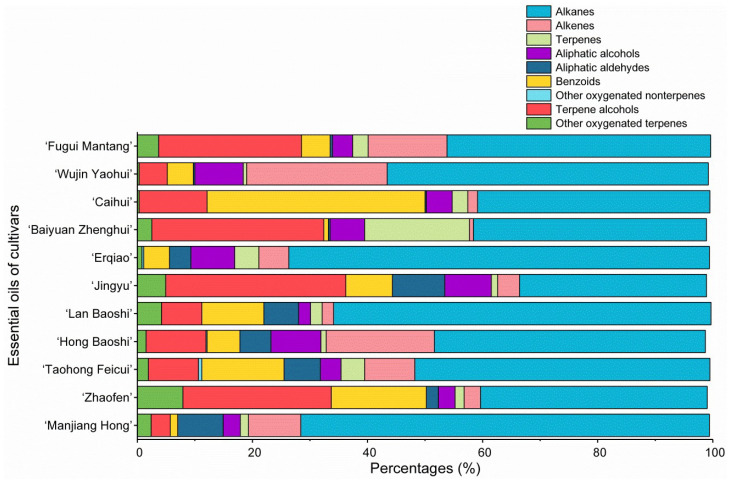
Group chemical components of flower essential oils of the eleven woody peony cultivars (*Paeonia × suffruticosa* Andr.).

Typical gas chromatograms of the essential oils of the eleven cultivars are given in Figure 2. A total of 105 compounds were identified, which accounted for 98.7–99.7% of total composition (Table 2). For individual cultivars, the compound numbers varied from 47 (‘Baiyuan Zhenghui’, ‘Zhaofen’ and ‘Manjiang Hong’) to 63 (‘Jingyu’). The essential oil components belong to alkanes, alkenes, terpenes, aliphatic alcohols, aliphatic aldehydes, benzoids, other oxygenated non-terpenes, terpene alcohols and other oxygenated terpenes (Figure 3).

The alkanes included tricosane, nonadecane, pentadecane, heptadecane, pentacosane and heneicosane, which were present in all the essential oils with varied percentages. Particularly, ‘Erqiao’, ‘Manjiang Hong’ and ‘Lan Baoshi’ essential oils contained alkanes at 65.6–73.1%. Interestingly, almost all of the major alkanes possessed an odd number of carbons, whereas alkanes having an even number of carbons were minor components (<2.5%). Branched alkanes were also found in the present study. Interestingly, the methyl was almost always attached to position 2 of the *n*-alkane main chain with the even carbon numbers (2-methylhexadecane, for example), and position 3 of the *n*-alkane main chain with the odd carbon numbers (3-methyltricosane, for example).

As for the alkenes, the essential oils of ‘Wujin Yaohui’ and ‘Hong Baoshi’ contained medium levels of this class (24.4% and 18.8%, respectively), mainly represented by 6,9-heptadecadiene (15.5% and 12.4%, respectively) and *trans*-8-heptadecene (4.9% and 4.0%, respectively). It was noted that the two cultivars possessed balanced ratios of alkane to alkene (2.3:1 and 2.5:1, respectively), while most of the others possessed larger ratios of alkane to alkene (for ‘Baiyuan Zhenghui’, it reached 57.9:1).

Terpene hydrocarbons were basically at low percentages. Only ‘Baiyuan Zhenghui’ essential oil presented a medium content (18.2%), which was mainly attributed to germacrene D (10.5%) and *trans*-β-ocimene (6.7%). In contrast, the percentages of germacrene D and *trans*-β-ocimene were much lower or not detected in other cultivars. In a recent report, the essential oil of *Pogostemon plectranthoides* from India presents chemotype of *trans*-β-ocimene—germacrene D—*trans*-β-guaiene [20]. High levels of germacrene D are usually accompanied by cadinane and muurolane sesquiterpenoids [21]. Interestingly, δ-cadinene and τ-muurolol were uniquely present in ‘Baiyuan Zhenghui’ essential oil.

Oxygenated non-terpenes possessed more variance in percentages. ‘Caihui’ contained the highest level of benzoids (37.9%), which was followed by ‘Zhaofen’ (16.5%) and ‘Taohong Feicui’ (14.3%). This was mainly attributed to phloroglucinol trimethyl ether, hydroquinone dimethyl ether and 4,7-dimethylbenzofuran. Distinctly, ‘Hong Baoshi’ contained benzeneethanol (4.0%). As for aliphatic alcohols (2.1–8.7%), the main components were *cis*-3-hexen-1-ol (1.2–4.2%) and 1-hexanol (0.7–3.7%) which were present in all of the essential oils. Other minor ones were *cis*-9-tridecen-1-ol, 2-heptadecanol, 1-eicosanol, 1-nonanol, 2-pentadecanol, 1-decanol (only in ‘Erqiao’), *cis*-3-nonen-1-ol (only ‘Taohong Feicui’), 2-hexadecanol (only ‘Wujin Yaohui’) and 1-octadecanol (only ‘Hong Baoshi’). As for aliphatic aldehydes (0.2–9.1%), nonanal was the main component.

Terpene alcohols occurred mainly in the essential oils of ‘Jingyu’, ‘Fugui Mantang’, ‘Baiyuan Zhenghui’ and ‘Zhaofen’ (24.8–31.3%), most of which were monoterpene alcohols. In particular, citronellol was mainly in the essential oils of ‘Zhaofen’ (25.8%), ‘Jingyu’ (23.1%) and ‘Baiyuan Zhenghui’ (21.6%). Geraniol was mainly in ‘Fugui Mantang’ (13.0%). Other terpene alcohols were nerol, *cis*-linalool oxide furan, *trans*-linalool oxide furan, linalool, α-terpineol and τ-muurolol. Terpene alcohols are valued for they play an important role in the quality profile of essential oils [22].

Other oxygenated terpenes included terpene esters, oxides, aldehydes, ketones, acids and derivatives, which occurred at relatively low levels. Citronellyl acetate occurred in the essential oils of ‘Zhaofen’ (3.9%), ‘Manjiang Hong’ (1.4%) and ‘Jingyu’ (1.0%). Some other citronellyl esters, namely citronellyl isobutyrate, citronellyl 2-methylbutanoate and citronellyl 3-methylbutanoate, occurred uniquely in the ‘Zhaofen’ essential oil (all < 1.0%). One of the reasons for the low level of esters and high level of alcohols might be hydrolysis that possibly occurred during hydro-distillation [23]. The *cis*-rose oxide and *trans*-rose oxide occurred only in ‘Jingyu’ (1.3% and 0.4%, respectively) and ‘Zhaofen’ (0.7% and 0.3%, respectively). Neral and geranial coexisted in ‘Fugui Mantang’ (1.2% and 1.4%, respectively), to which neral was unique. Prenylacetone, geranyl acetone, hexahydrofarnesyl acetone and farnesyl acetone were all at low levels (<1.0%). Geranic acid mainly occurred in the essential oils of ‘Lan Baoshi’ (3.5%), ‘Baiyuan Zhenghui’ (1.4%) and ‘Hong Baoshi’ (1.0%). It was noted that most of the oxygenated terpenes belonged to acyclic types, indicating that the terpene biosynthesis remained at initial steps of biosynthetic pathway.

### 2.3. Multivariate Analysis Results

**Figure 4:**
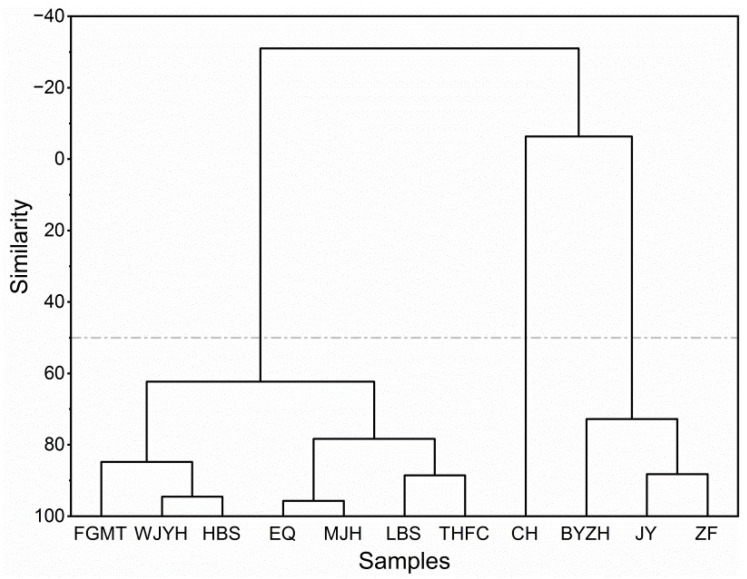
Dendrogram from HCA of flower essential oils of the eleven woody peony cultivars (*Paeonia × suffruticosa* Andr.). Clustering is based on similarity under Ward linkage and squared Euclidean distance. For codes of cultivar names, see Table 1.

The hierarchical cluster analysis (HCA) dendrogram clearly assigned the essential oils into three clusters (Figure 4). Cluster I only consisted of ‘Caihui’, which was distinct from others. Cluster II consisted of ‘Baiyuan Zhenghui’, ‘Jingyu’ and ‘Zhaofen’. Cluster III consisted of the rest of the cultivars.

**Figure 5:**
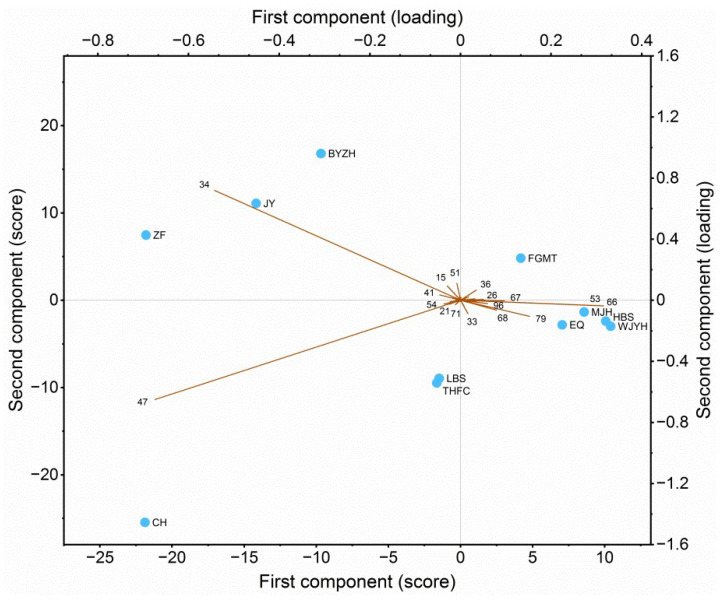
Score and loading plots from PCA of flower essential oils of the eleven woody peony cultivars (*Paeonia × suffruticosa* Andr.). Plots are presented along the first two principal components (74.1% of total variance). For codes of cultivar names, see Table 1.

As for principal component analysis (PCA) (Figure 5), the chemical variance was presented with the first two principal components, which in sum explained 74.1% of the total variance (PC1 39.8%, PC2 34.3%). PCA was generally consistent with HCA in the sample grouping. It gave further information on the correlation of each cluster with corresponding characteristic compounds. Cluster I consisting of ‘Caihui’ was located at the lower left corner. It was highly correlated with phloroglucinol trimethyl ether. Cluster II consisting of ‘Jingyu’, ‘Zhaofen’ and ‘Baiyuan Zhenghui’ was located at the upper left corner. This cluster was correlated with citronellol. Interestingly, ‘Zhaofen’ and ‘Jingyu’ exhibited not only high levels of citronellol but also low levels of other rose-related aroma compounds (citronellyl esters, *cis*-rose oxide and *trans*-rose oxide). Therefore, they might serve as alternatives to rose-type essential oils. Cluster III consisting of the rest of cultivars was somewhat diverse. ‘Fugui Mantang’ located at the upper right corner was mainly correlated with geraniol, hydroquinone dimethyl ether and *trans*-8-heptadecene. ‘Lan Baoshi’ and ‘Taohong Feicui’ were correlated with phloroglucinol trimethyl ether, nonadecane and nerol. Others were correlated with pentadecane, 6,9-heptadecadiene, nonadecane and pentadecane.

### 2.4. Target Network Characteristics

Altogether 45 essential oil compounds were retained after excluding those lower than 1.0% in all samples. Further, drug-likeness screening afforded 20 compounds with zero violation to Lipinski’s rule (Table S1 in Supplementary Materials). Interestingly, all the 20 compounds showed predictive blood–brain barrier (BBB) permeation. They were all oxygenated compounds, except *trans*-β-ocimene which is a monoterpene hydrocarbon. Specifically, they were oxygenated benzoids (phloroglucinol trimethyl ether, hydroquinone dimethyl ether, 4,7-dimethylbenzofuran and benzeneethanol), short chain aliphatic alcohols and aldehydes (*cis*-3-hexen-1-ol, 1-hexanol, nonanal and hexanal), terpene alcohols (citronellol, geraniol, *cis*-linalool oxide furan, *trans*-linalool oxide furan, nerol and linalool) and other oxygenated terpenes (citronellyl acetate, geranic acid, *cis*-rose oxide, geranial and neral).

**Figure 6:**
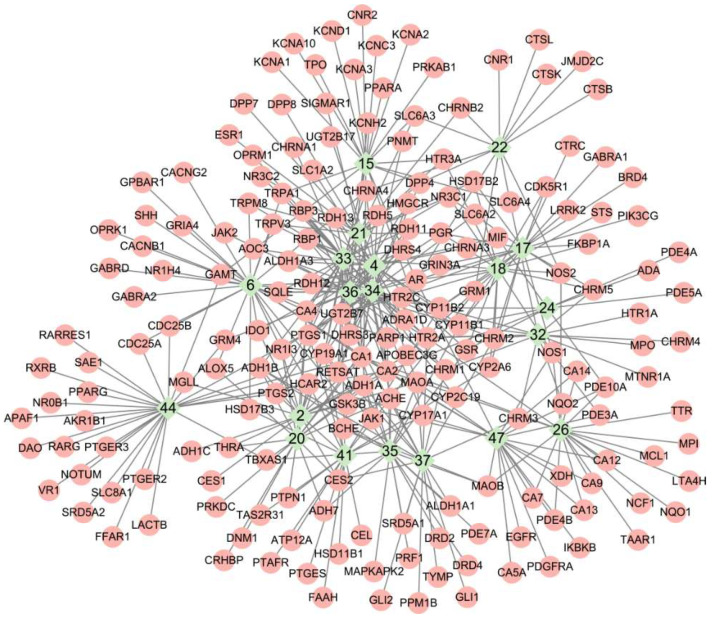
Compound target network of 20 essential oil compounds of woody peony flowers and 190 potential targets. Compounds are represented by green diamonds noted with numbers (see Table 2). Targets are represented by pink circles noted with standard gene names.

Target prediction for the 20 compounds afforded 190 potential targets (Table S2 in Supplementary Materials). The compound target network consisted of 210 nodes (compounds and targets) and 420 edges (compound target connections) (Figure 6). The top ranked compounds were nerol, citronellol, geraniol, geranic acid, *cis*-3-hexen-1-ol and 1-hexanol, which were the most important in the network (Table 3). They also constituted the major components of the essential oils.

**Figure 7:**
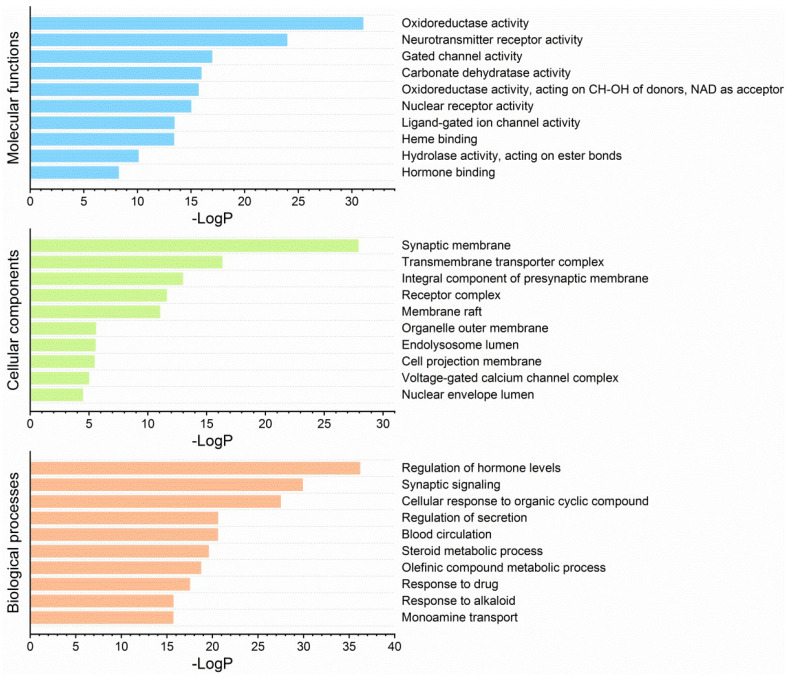
Enrichment analysis on GO molecular functions, cellular components and biological processes that demonstrates the representative top 10 enriched terms.

**Figure 8:**
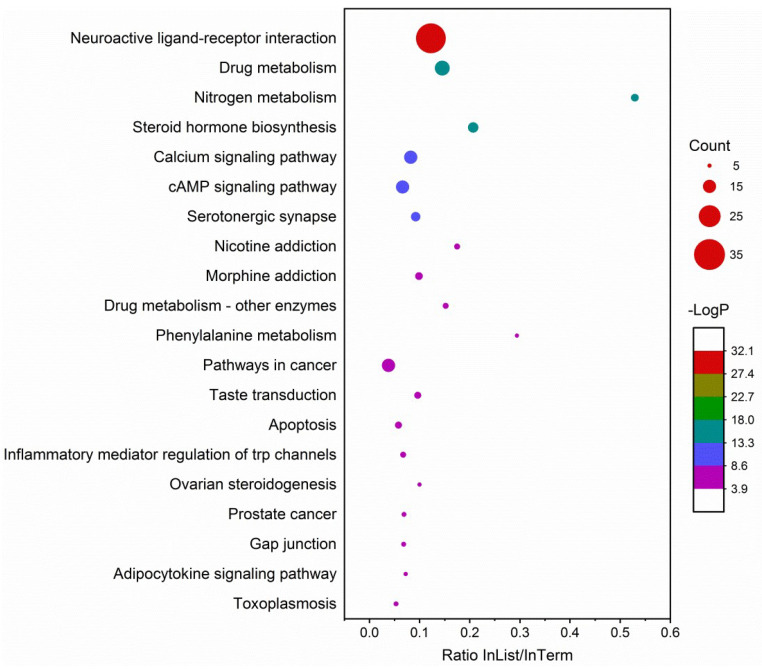
Enrichment analysis on KEGG pathways that demonstrates the representative top 20 enriched terms. Count, the number of input genes that fall into this term; −Log P, the minus of *p*-value in log base 10; InList/InTerm ratio, the ratio of the gene count in the list that hit the term to the gene count of the total genome of this term.

**Figure 9:**
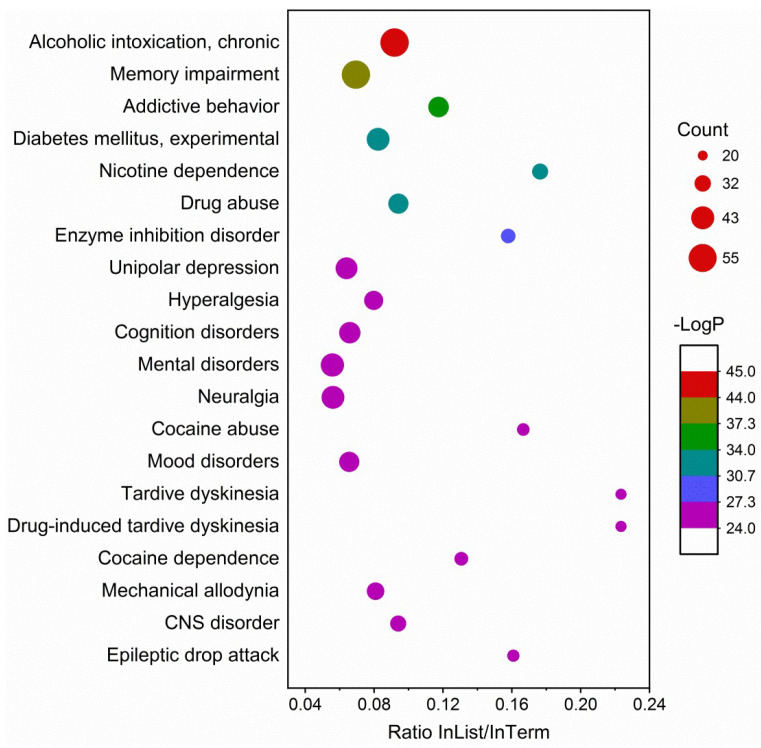
Enrichment analysis on DisGeNET that demonstrates the representative top 20 enriched diseases. Count, the number of input genes that fall into this term; −Log P, the minus of *p*-value in log base 10; InList/InTerm ratio, the ratio of the gene count in the list that hit the term to the total gene count of this term.

Enrichment analyses included GO, KEGG and DisGeNET. GO molecular functions were mainly enriched in oxidoreductase and neurotransmitter receptor activities. GO cellular components were enriched in synaptic membrane and transmembrane transporter complex. GO biological processes were enriched in the regulation of hormone levels, synaptic signaling and cellular response to organic cyclic compounds (Figure 7). KEGG pathways were predominantly enriched in neuroactive ligand–receptor interaction, the log_10_P (−32.08) of which was much lower than others (Figure 8). Others included drug metabolism, nitrogen metabolism and steroid hormone biosynthesis. DisGeNET diseases were mainly enriched by chronic alcoholic intoxication, memory impairment and addictive behavior (Figure 9).

**Figure 10:**
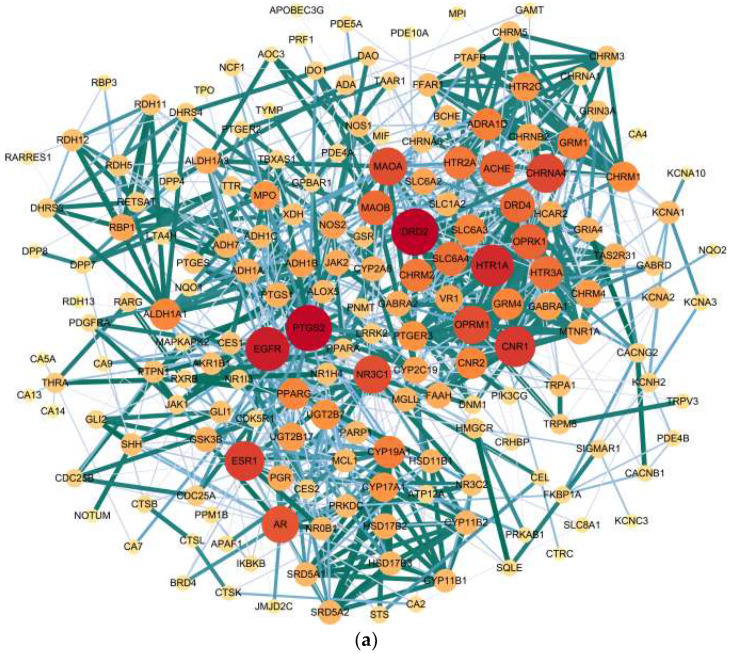

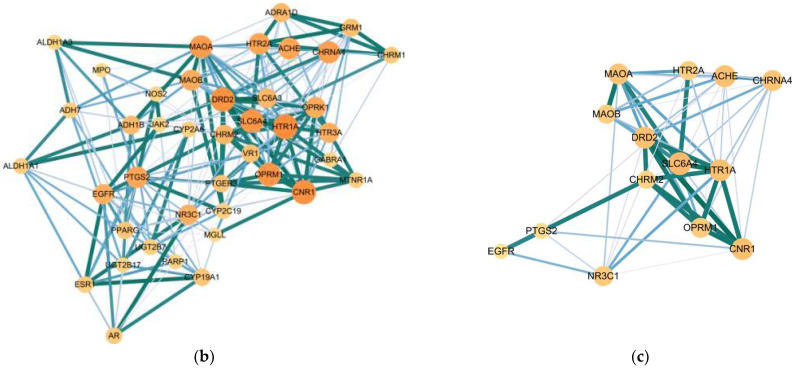
PPI network of the potential targets of essential oil compounds of woody peony. (**a**) Total network consisting of all connected nodes (183 targets); (**b**) Subnetwork consisting of 41 targets after first filtering applied; (**c**) Core network consisting of 14 targets after second filtering applied. The size and color of each node is representative of its degree value within the present network. The line width and color of each edge is representative of the combined interaction score.

The protein—protein interaction (PPI) network of the 190 targets identified 183 connected nodes (targets) and 992 edges (interactions) (Figure 10a; Table S3 in Supplementary Materials). The first filtering was applied to retain nodes with degree, closeness centrality and betweenness centrality, all above average (10.84, 0.3669 and 9.842 × 10^−3^, respectively), resulting in a subnetwork of 41 targets (Figure 10b). Topological parameters of this subnetwork were recalculated and the second filtering was applied to retain nodes with new degree, closeness centrality and betweenness centrality, all above average (10.83, 0.5435 and 2.208 × 10^−2^, respectively), which afforded 14 core targets (Figure 10c).

The final ranking of the 14 targets in this core network (Table 4) was SLC6A4 (sodium-dependent serotonin transporter), CNR1 (cannabinoid receptor 1), ACHE (acetylcholinesterase), HTR1A (5-hydroxytryptamine receptor 1A), DRD2 (dopamine D2 receptor), MAOA (monoamine oxidase type A), CHRNA4 (neuronal acetylcholine receptor subunit alpha-4), NR3C1 (glucocorticoid receptor), OPRM1 (mu-type opioid receptor) and HTR2A (5-hydroxytryptamine receptor 2A), MAOB (monoamine oxidase type B), CHRM2 (muscarinic acetylcholine receptor M2), PTGS2 (prostaglandin G/H synthase 2) and EGFR (epidermal growth factor receptor).

## 3. Discussion

The eleven cultivars of the woody peony (*P. × suffruticosa*) investigated in this study were representative ones that are widely cultivated in the Central Plains of China, especially the Luoyang district. They belong to different color series of petals due to varied contents of anthocyanins [24]. In the present study, they were grown under similar ecological and climatic conditions. Moreover, the harvest and extraction conditions were almost constantly controlled. Therefore, the variances in the essential oil yields were mainly attributed to genotypes. On the other hand, the yields obtained herein were lower than that of the dried flower buds of *P.* × *suffruticosa* (0.79%, cultivar not specified) [6]. They were also lower than the reported yields achieved by supercritical CO_2_ extraction from the dried petals of eleven *P.* × *suffruticosa* cultivars (0.81–1.09%, including ‘Jingyu’ 0.93% and ‘Erqiao’ 0.95%, which were investigated in the present study as well) [7]. Meanwhile, they were comparable to or lower than those of the flower essential oils of eight other *P.* × *suffruticosa* cultivars investigated in our previous study (0.28–0.93%) [8]. In contrast, they were higher than the reported yields of the fresh petals of two woody peony cultivars (0.09% and 0.10%, possibly on fresh basis) [5] and dried petals of *P.* × *suffruticosa* (0.66 mg/g, cultivar not specified) [4]. The yield variances between the present study and literature data can be attributed to genotypes, ecological and climatic conditions [10,12], harvest or postharvest factors, and, more importantly, extraction methods [13,14]. Indeed, application of innovative techniques improves the extraction efficiency in terms of time and energy consumption, as well as production yield and quality of essential oils [4,14,23]. Further studies are needed for the evaluation and comparison of green and innovative techniques with the same woody peony flower materials.

As for the chemical composition of woody peony essential oils, there are remarkable differences between this study and the literature, and between different literature reports. Our previous studies have focused on hydrolate extracts of some other *P.* × *suffruticosa* cultivars which possess higher percentages of oxygenated compounds (87.4–99.8%). Specifically, they show a predominance of benzeneethanol (48.0–79.5%), phloroglucinol trimethyl ether (50.2–72.8%), citronellol (30.7–57.2%) or balanced chemical types [8,9]. Han and Bhat [6] reported on hydro-distilled essential oil of *P.* × *suffruticosa* dried flower buds, which were characterized by *n*-alkanes (28.6%), methyl isobutyl ketone (0.8%), benzaldehyde (0.6%), furfural (0.4%) and hexadecanoic acid methyl ester (0.3%). Zhang et al. [4] reported on microwave-assisted steam-distilled essential oil of *P.* × *suffruticosa* petals (cultivar not specified) from East China, which were characterized by tetracosane (24.9%), docosane (20.7%), hexacosane (16.6%), α-terpineol (3.3%), geraniol (2.6%), linalool (1.2%), 8-heptadecene (1.4%) and *cis*-linalool oxide (1.0%). They also found that the chemical composition altered with specific distillation methods. In a study on supercritical CO_2_-extracted petal essential oils of eleven *P.* × *suffruticosa* cultivars and three wild *Paeonia* species from Northwest China, the main components out of the 163 identified ones were linalool oxide, (*Z*)-5-dodecenyl acetate, nonadecane, (*Z*)-5-nonadecene, heneicosane, phytol and ethyl linoleate. ‘Jingyu’ and ‘Erqiao’ essential oils have also been reported in this literature study [7]. However, their identified chemical compositions in the present study were quite different. In the present study, ‘Jingyu’ essential oil possessed much higher citronellol content (23.1% vs. 0.1%). For ‘Erqiao’ essential oil, the predominant higher fatty acid esters in the literature report were not found in the present study. Recently, Wu et al. [5] reported that the essential oils of two woody peony cultivars from Northwest China were different in chemical composition. ‘ZiBan’ is represented by alcohols, alkanes and acids, whereas ‘FengDan’ is represented by aldehydes, alcohols and terpenes.

As for the multivariate analysis of woody peony flower essential oils, literature data are scarce. Our previous study focused on hydrolate extracts of woody peony flowers. The PCA results revealed that the cultivar ‘Linghua Zhanlu’ was most distinct among the ten cultivars, due to the occurrence of geraniol, 6,9-heptadecadiene, 2-heptanol, pentadecane and *cis*-3-nonen-1-ol [9]. In another study involving eight other cultivars, the clustering of hydrolate samples was somewhat not typical [8]. Zhang et al. [7] reported four clusters from supercritical extracted petal essential oils of eleven *P.* × *suffruticosa* cultivars and three wild *Paeonia* species, which were represented by ethyl linoleate, (*Z*)-5-dodecenyl acetate, octadecanal and linalool oxide, respectively. In the present study, three clusters representative of phloroglucinol trimethyl ether, citronellol and mixed chemotypes emerged from the HCA and PCA, which were different from literature data. Even for the two cultivars ‘Jingyu’ and ‘Erqiao’, which were also reported on in the literature [7], their chemotypes in the present study were different. It is reported that the chemical type variances of essential oils are due to genotypes [11,12], geographical and climatic conditions [10], harvest and postharvest factors and extraction methods [13,14].

The 20 compounds that survived drug-likeness screening should be stressed in terms of potential activities of woody peony essential oils, particularly the six key compounds (nerol, citronellol, geraniol, geranic acid, *cis*-3-hexen-1-ol and 1-hexanol) summarized from the compound target network. They were mostly oxygenated compounds, which are recognized to be more important than hydrocarbons [8,25]. Literature reports suggest central nervous system (CNS) activities for most of the 20 compounds. Nerol possesses an anxiolytic effect in mice [26]. Geraniol shows neuroprotective effects against zinc oxide nanoparticles in rats [27]. Citronellol possesses anti-nociceptive, anti-anxiety and anticonvulsant effects with low toxicity [28]. Geranic acid is the sequential oxidative product of geraniol and geranial, which is less odor-active but acts as enhancer for geraniol-related oxygenated terpenes [29]. The *cis*-3-hexen-1-ol and 1-hexanol possess fresh green leaf aroma [30]. A mixture of *cis*-3-hexen-1-ol and *trans*-2-hexenal attenuates behavioral and stress responses induced by olfactory and noxious stimuli in rats [31]. In addition, 1-hexanol, as well as other short chain alcohols, acts as a CNS depressant through the inhibition of NMDA (*N*-methyl-D-aspartate) gated currents [32]. Besides the six key compounds, others of the 20 compounds should not be disregarded. Linalool, as well as linalool abundant *Lavandula angustifolia* essential oil, counteracts social aversion induced by social defeat [33]. Linalool oxide furan has an anxiolytic effect upon inhalation [34]. Citronellyl acetate possesses an anti-nociceptive effect in mice, possibly involving TRPV1, TRPM8 and ASIC [35]. Rose oxide has an antidepressant activity, probably through the serotonergic pathway that involves the HTR1A receptor [36]. Citral, the mixture of geranial and neral, presents sedative and motor relaxant effects in mice [37]. In a recent report, citral exhibits anticonvulsant effect in zebrafish [38]. Phloroglucinol trimethyl ether, as well as similar oxygenated benzoids, constitutes typical flower volatiles of some varieties of *Rosa odorata* and *R. chinensis*. It presents a sedative effect and is used as perfume ingredients [39]. Benzeneethanol, a benzoid alcohol with pleasant aroma characteristic of rose flowers, has a sedative activity via inhalation [40]. The 4,7-dimethylbenzofuran also occurs in the volatiles of woody peony seed oil, which possesses aromas of grass, slight bitterness and fragrance [41].

Further enrichment analysis of the potential targets clearly indicated that the top enriched terms in GO, KEGG and DisGeNET were mostly related to the CNS, particularly neuroactive ligand–receptor interaction in KEGG pathways, and chronic alcoholic intoxication, memory impairment and addictive behavior in DisGeNET diseases. More importantly, PPI analysis indicated that 11 of the 14 core targets were CNS-related. Specifically, SLC6A4, HTR1A and MAOA are targets for antidepressants. CNR1 and OPRM1 are targets for analgesics. DRD2 and HTR2A are targets for antipsychotics. ACHE is the target for treatment of Alzheimer’s disease. CHRNA4, MAOB and CHRM2 are targets for the treatment of Parkinson’s disease [42,43]. Indeed, all the 20 compounds that survived drug-likeness screening showed predictive BBB permeation. Besides, when essential oil compounds pass through the BBB, they possibly affect the CNS [44]. Further, some essential oils and their main constituents are active on the CNS [33]. As for the other three core targets, NR3C1 and PTGS2 are targets for steroidal and non-steroidal anti-inflammatory agents, respectively. EGFR is the target for treatment of non-small-cell lung cancer with gene mutants [42,43].

The findings that came from this study are important and informative for developing the practical applications of the woody peony flower essential oils. The chemotype identification provides useful information on their specific uses in flavors, fragrances and cosmetics. For example, the ‘Zhaofen’ and ‘Jingyu’ essential oils may serve as alternatives to rose-type essential oils, due to high levels of citronellol as well as low levels of other rose-related aroma compounds. This study also gives instructions on the selection of specific chemo-cultivars of woody peony for cultivation and essential oil production. Moreover, the results of target network analysis suggest that the woody peony essential oils have a potential importance in aromatherapy, especially for alternative treatment of CNS disorders. Importantly, natural fragrant compounds possess relatively low neurotoxicity and are basically safe [44]. Besides, treatment for CNS disorders nowadays has more or less side effects [28]. Therefore, woody peony flower essential oils can be developed for aromatherapy applications. Nevertheless, further studies are needed on their in vivo effects and specific mechanisms.

## 4. Materials and Methods

### 4.1. Materials and Regents

Flower materials of *P.* × *suffruticosa* Andr. were collected in April 2019 from the Garden of National Flower of China. The location coordinate was 112.45° E and 34.65° N with an altitude of 140 m, Luoyang, Henan Province, China. Eleven representative cultivars were collected, namely ‘Fugui Mantang’, ‘Wujin Yaohui’, ‘Caihui’, ‘Baiyuan Zhenghui’, ‘Erqiao’, ‘Jingyu’, ‘Lan Baoshi’, ‘Hong Baoshi’, ‘Taohong Feicui’, ‘Zhaofen’ and ‘Manjiang Hong’ (Table 1). Authentication of plant materials was performed by Dr. Gaoming Lei, Department of Pharmaceutical Sciences, Henan University of Science and Technology. Voucher specimens numbering PS190412-01~PS190412-11 are deposited at Herbarium of Department of Pharmaceutical Sciences, Henan University of Science and Technology. Analytical standard of C7–C30 saturated alkanes with individual component of 1000 μg/mL in hexane (ID 49451-U, lot LRAC0353) was purchased from Sigma-Aldrich, Laramie, WY, USA. Purified water was produced by Wahaha Group Co., Ltd., Hangzhou, China. The *n*-hexane, sodium chloride, anhydrous sodium sulfate and other chemical reagents were of analytical grade.

### 4.2. Hydro-Distillation

Fresh flowers of woody peony were hydro-distilled in a Clevenger-type apparatus (Changsheng Apparatus for Science and Education, Hangzhou, China). The essential oil was trapped in *n*-hexane on top of a water layer inside the receiver of the apparatus. The hydro-distillation was performed in triplicate. The organic layer was collected and dried with anhydrous sodium sulfate. It was evaporated to afford the essential oil which was stored in 5 °C prior to chromatographic analysis. The yield was measured as the mass percent of the essential oil relative to the dried basis of fresh flowers. The water content of fresh flowers which was determined through distillation with toluene was used for calculation.

### 4.3. GC-MS and GC-FID Analysis

GC-MS was performed on 6890N GC with 5975 inert MSD (Agilent Technologies, Santa Clara, CA, USA) using HP-5ms column (length of 30 m, inner diameter of 0.25 mm, film thickness of 0.25 μm, Agilent Technologies, Santa Clara, CA, USA). The essential oil was diluted in hexane, 1.0 μL of which was injected to the chromatograph. The split mode was selected and split ratio was 20:1. The injector temperature was 250 °C. The carrier gas was helium and column flow was set at 1.0 mL/min. The initial temperature of the column was set at 50 °C. After injection, it was immediately raised to 200 °C at 3 °C/min, further to 240 °C at 10 °C/min, and kept thermostatically for 15 min. For the essential oil samples, there was a post run set at 250 °C for 2 min. For the C7–C30 saturated alkanes standard, after being kept at 240 °C for 15 min, the column temperature was further raised to 250 °C at 10 °C/min and then kept at 250 °C for 10 min. The MSD transfer line was set at 280 °C. Full scan mode was used at the range of 29–400 amu. The MS quadrupole was set at 150 °C and the ion source was 230 °C. The ion energy was 69.9 eV. GC-FID was performed on 6890N GC with FID (Agilent Technologies, Santa Clara, CA, USA) using HP-5ms column (30 m × 0.25 mm id, film thickness 0.25 μm, Agilent Technologies, Santa Clara, CA, USA). Analytical conditions were as follows: injection volume of 1.0 μL, carrier gas of helium, split mode, split ratio of 20:1, injector temperature of 250 °C, constant flow mode, column flow of 1.0 mL/min, FID temperature of 280 °C. The temperature program of the column was the same as that of GC-MS.

The essential oil components were identified taking into account mass spectra and retention indices. Experimental mass spectrum of each peak was searched against the reference mass spectra database NIST05a. The experimental retention index of each peak determined with C7–C30 saturated alkanes standard was compared with literature values [8,9,18,19]. The percentage of the individual components was calculated using the method of peak area integration based on FID response.

### 4.4. Multivariate Analysis

The total essential oil composition from the eleven woody peony cultivars was subjected to multivariate analysis of HCA and PCA. The data for analysis contained percentages of individual components of all the eleven cultivars. For HCA, Ward linkage and squared Euclidean distance was used. For PCA, the covariance matrix was used. The analysis was performed using R software [45].

### 4.5. Target Network Analysis

Essential oil components with percentages lower than 1.0% in all samples were not considered, and were excluded. Retained compounds were submitted to PubChem (https://pubchem.ncbi.nlm.nih.gov/, accessed on 30 March 2021) to retrieve their chemical information [46]. The compounds were submitted to SwissADME (http://www.swissadme.ch/, accessed on 2 April 2021) for drug-likeness screening under the criterion of zero violation to Lipinski’s rule [47]. Compounds that survived were subjected to target prediction in *Homo sapiens*. To make predictive results as accurate and inclusive as possible, three online tools were utilized in a complementary way. They were SwissTargetPrediction [48] (http://swisstargetprediction.ch/, accessed on 6 April 2021; Parameters: probability > 0, top 10), ChemMapper [49] (http://lilab-ecust.cn/chemmapper/index.html, last accessed on 1 May 2021; Parameters: 3D similarity by SHAFTS method [50], BindingDB database [51], similarity threshold 1.2, top 10) and BATMAN [52] (http://bionet.ncpsb.org.cn/batman-tcm/, accessed on 7 April 2021; Parameters: score cutoff 80, adjusted *p*-value cutoff 0.05, top 10). Afterwards, the predicted targets were combined and integrated with standard gene names using UniProt (https://www.uniprot.org/, accessed on 28 May 2021) [53]. The compound target network was visualized and analyzed by Cytoscape software [54].

Enrichment analysis on GO [55,56], KEGG [57] and DisGeNET [58] was performed on Metascape (https://metascape.org/gp/index.html#/main/step1, accessed on 18 June 2021) [59]. Parameters were: input as *H. sapiens*, minimum overlap 3, *p*-value cutoff 0.01 and minimum enrichment factor 1.5. The list of potential targets was also submitted to STRING (https://cn.string-db.org/, accessed on 4 June 2021) [60] to evaluate the PPI network. Parameters were: type of full network, active interaction sources as text mining, experiments, databases, co-expression, neighborhood, gene fusion and co-occurrence, minimum required interaction score as medium confidence at 0.40. The PPI network of connected nodes (the disconnected nodes were not considered in this step) was visualized and analyzed by Cytoscape software [54]. To identify the core targets, the entire network was refined in two consecutive steps, each of which involved retaining nodes with degree, closeness centrality and betweenness centrality all above average.

### 4.6. Other Statistics

Other statistics were calculated with R software [45]. ANOVA was performed to evaluate the statistical significance of data variation. Post-hoc test with Tukey’s method was used for multiple comparisons. The significance level was set as 0.05 unless otherwise noted.

## 5. Conclusions

Eleven representative woody peony cultivars (*Paeonia* × *suffruticosa* Andr.) were investigated for the chemical diversity of flower essential oils and potential target network of the components. The chemical compositions of the essential oils exhibited variances, suggesting that they were affected by the genotypes. HCA and PCA analyses gave three distinct clusters representative of phloroglucinol trimethyl ether (‘Caihui’), citronellol (‘Jingyu’, ‘Zhaofen’ and ‘Baiyuan Zhenghui’) and mixed (the rest of the cultivars) chemotypes. Interestingly, ‘Zhaofen’ and ‘Jingyu’ exhibited not only high levels of citronellol but also low levels of other rose-related aroma compounds, which allowed them to serve as alternatives to rose-type essential oils. Drug-likeness screening gave 20 essential oil compounds. Interestingly, they all exhibited predictive BBB permeation. The compound target network demonstrated six key compounds, namely, nerol, citronellol, geraniol, geranic acid, *cis*-3-hexen-1-ol and 1-hexanol, which should be especially stressed in terms of the potential activities of the woody peony essential oils. Enrichment analysis on GO, KEGG and DisGeNET indicated that the top enriched terms were mostly related to CNS. PPI analysis resulted in 14 core targets, 11 of which were CNS-related, including those for antidepressants, analgesics, antipsychotics, and anti-Alzheimer’s and anti-Parkinson’s agents. The findings that came from this work provide useful information for the selection of the specific chemo-cultivars of woody peony for cultivation and essential oil production. They also demonstrate the potential importance of woody peony flower essential oils in aromatherapy, especially for the alternative treatment of CNS disorders. Further studies are needed on their in vivo effects and specific mechanisms.

## Figures and Tables

**Table 1:** Hydro-distillation yields of flower essential oils of the eleven woody peony cultivars (*Paeonia* × *suffruticosa* Andr.).

Cultivar Name	Cultivar Name Translated	Code	Specimen No.	EO Yield (*w*/*w*%) ^a^
‘Fugui Mantang’	Full of wealth and rank	FGMT	PS190412-01	0.14 ± 0.05 ^B^
‘Wujin Yaohui’	Dark gold that glitters	WJYH	PS190412-02	0.13 ± 0.02 ^B^
‘Caihui’	Colorful painting	CH	PS190412-03	0.17 ± 0.03 ^A,B^
‘Baiyuan Zhenghui’	Competing for shining in the great flower garden	BYZH	PS190412-04	0.17 ± 0.04 ^A,B^
‘Erqiao’	Two distinct beauties	EQ	PS190412-05	0.18 ± 0.05 ^A,B^
‘Jingyu’	In honor of Horticulturist Jingyu Sun	JY	PS190412-06	0.11 ± 0.01 ^B^
‘Lan Baoshi’	Blue gems	LBS	PS190412-07	0.19 ± 0.06 ^A,B^
‘Hong Baoshi’	Red gems	HBS	PS190412-08	0.16 ± 0.02 ^A,B^
‘Taohong Feicui’	Peach red with flying emerald green	THFC	PS190412-09	0.25 ± 0.04 ^A^
‘Zhaofen’	Pink flowers from Zhao (horticulturist in Qing Dynasty)	ZF	PS190412-10	0.13 ± 0.02 ^B^
‘Manjiang Hong’	Red floating plants all over the river	MJH	PS190412-11	0.16 ± 0.03 ^A,B^

^a^ Yield of flower essential oil (*w*/*w*% on dry basis) presented as mean value with standard deviation of triplicate distillations. Values that did not share a capital letter were significantly different according to the ANOVA with Tukey’s method (*p* < 0.05).

**Table 2:** Chemical composition of flower essential oils of the eleven woody peony cultivars (*Paeonia* × *suffruticosa* Andr.).

No.	RI_det_ ^a^	RI_ref_ ^b^	Compound ^c^	FGMT ^d^	WJYH	CH	BYZH	EQ	JY	LBS	HBS	THFC	ZF	MJH
1	-	800	Octane	0.1	0.1	0.7	0.1	0.6	2.3	0.6	0.4	0.9	0.3	1.3
2	802	801	Hexanal	0.2	0.3	-	0.3	0.4	-	1.0	1.0	0.9	0.3	1.2
3	852	854	*trans*-2-Hexenal	-	-	-	-	-	0.2	-	0.2	-	-	-
4	860	858	*cis*-3-Hexen-1-ol	1.4	2.1	2.3	2.9	3.2	4.2	1.2	4.0	1.9	1.7	1.5
5	870	868	1,4-Dimethylbenzene	0.1	-	-	0.2	0.2	-	-	0.3	tr	0.1	0.1
6	873	871	1-Hexanol	1.7	3.7	2.1	2.9	2.5	2.9	0.7	3.6	1.0	1.2	0.9
7	882	882	2,6-Dimethyl-1,5-heptadiene	0.3	-	-	0.4	-	0.8	0.3	-	-	0.3	-
8	899	900	Nonane	-	-	-	-	-	0.2	-	-	-	-	-
9	903	902	Heptanal	-	-	0.2	-	0.1	0.5	0.2	0.2	-	-	0.3
10	931	932	α-Pinene	0.2	-	-	-	-	0.5	0.2	-	-	-	-
11	989	989	Prenylacetone	0.3	-	-	0.3	-	0.4	-	-	-	-	-
12	992	993	2-Pentylfuran	-	-	-	-	-	-	-	-	0.3	-	-
13	1005	1004	Octanal	-	-	-	-	-	0.2	-	-	-	-	-
14	1038	1037	*cis*-β-Ocimene	-	-	-	tr	-	-	-	-	-	-	-
15	1049	1048	*trans*-β-Ocimene	0.3	-	-	6.7	-	-	-	-	-	-	-
16	1050	1046	Benzeneacetaldehyde	-	-	-	-	-	-	-	tr	-	-	-
17	1075	1074	*cis*-Linalool oxide furan	1.2	0.2	2.0	1.5	-	1.6	0.7	1.1	1.2	-	0.4
18	1091	1088	*trans*-Linalool oxide furan	0.5	-	1.0	0.7	-	0.7	0.3	0.6	0.5	-	-
19	1099	1100	Undecane	-	0.3	0.4	0.5	0.7	0.2	-	0.2	-	-	0.2
20	1107	1104	Nonanal	-	-	-	-	3.2	7.2	4.8	3.8	5.4	1.8	6.4
21	1108	1106	Linalool	1.9	1.6	3.1	1.8	-	-	-	-	-	-	-
22	1113	1111	*cis*-Rose oxide	-	-	-	-	-	1.3	-	-	-	0.7	-
23	1128	1127	*trans*-Rose oxide	-	-	-	-	-	0.4	-	-	-	0.3	-
24	1130	1127	Benzeneethanol	-	-	-	-	-	-	-	4.0	0.4	-	-
25	1164	1160	*cis*-3-Nonen-1-ol	-	-	-	-	-	-	-	-	0.4	-	-
26	1171	1170	Hydroquinone dimethyl ether	4.1	2.7	0.4	-	2.5	-	-	-	-	0.2	-
27	1181	1176	1-Nonanol	-	-	-	-	0.5	0.9	-	-	-	-	-
28	1184	1180	Myrtanal	-	-	-	-	-	-	-	0.3	0.8	-	-
29	1199	1200	Dodecane	-	0.5	-	-	0.2	-	-	-	-	-	-
30	1202	1201	α-Terpineol	0.3	-	0.8	0.7	-	0.3	0.7	0.6	0.3	-	-
31	1208	1208	Decanal	-	-	-	-	-	0.3	-	-	-	-	-
32	1221	1220	4,7-Dimethylbenzofuran	0.4	0.6	0.5	0.6	1.5	0.7	0.9	0.6	1.0	0.6	0.7
33	1237	1230	Nerol	-	0.9	2.5	-	-	-	3.6	4.2	2.5	-	-
34	1239	1231	Citronellol	7.9	-	-	21.6	-	23.1	-	-	-	25.8	2.2
35	1246	1240	Neral	1.2	-	-	-	-	-	-	-	-	-	-
36	1264	1256	Geraniol	13.0	2.2	2.4	3.0	-	5.6	1.7	3.9	4.2	-	0.7
37	1276	1270	Geranial	1.4	-	-	0.8	-	0.2	-	-	-	-	-
38	1283	1275	1-Decanol	-	-	-	-	0.4	-	-	-	-	-	-
39	1299	1300	Tridecane	0.3	0.9	0.2	0.8	0.5	0.2	0.2	0.6	0.6	0.3	0.6
40	1327	1326	Geranic acid methyl ester	-	-	-	-	-	0.2	-	-	-	-	-
41	1355	1354	Citronellyl acetate	-	-	-	-	-	1.0	-	-	-	3.9	1.4
42	1386	1383	Geranyl acetate	-	-	-	-	-	0.3	-	-	-	-	-
43	1391	1385	*trans*-3-Tetradecene	-	-	-	-	0.2	-	-	-	-	-	-
44	1396	1393	Geranic acid	0.8	-	-	1.4	-	-	3.5	1.0	-	0.3	-
45	1399	1400	Tetradecane	1.3	1.1	0.7	1.6	0.9	-	2.2	2.0	0.4	0.8	1.4
46	1414	1407	Eugenol methyl ether	-	-	0.2	-	-	-	-	-	-	-	-
47	1423	1418	Phloroglucinol trimethyl ether	0.4	1.2	36.8	-	0.3	7.3	9.9	0.8	12.9	15.6	-
48	1457	1455	Geranyl acetone	-	-	0.1	-	0.2	0.4	0.2	-	0.3	0.2	0.3
49	1461	1462	2,6,10-Trimethyltridecane	0.3	0.4	0.5	0.2	1.0	1.0	0.9	0.3	0.8	1.1	1.2
50	1476	1475	1,14-Pentadecadiene *	-	0.2	-	-	-	-	-	-	-	-	-
51	1483	1481	Germacrene D	2.2	0.6	0.3	10.5	2.1	-	1.6	0.9	4.1	-	0.4
52	1484	1485	Citronellyl isobutyrate	-	-	-	-	-	-	-	-	-	0.4	-
53	1500	1500	Pentadecane	10.1	11.1	2.7	2.5	7.1	1.9	2.3	13.2	9.0	3.6	12.8
54	1510	1508	α-Farnesene	-	-	2.4	0.6	2.1	0.6	0.2	-	-	1.6	1.0
55	1526	1524	δ-Cadinene	-	-	-	0.4	-	-	-	-	-	-	-
56	1570	1570	3-Methylpentadecane	-	0.8	-	-	-	-	-	-	-	-	-
57	1571	1570	*cis*-9-Tridecen-1-ol *	0.4	-	-	-	0.3	-	-	0.7	0.3	-	0.6
58	1572	1572	Citronellyl 2-methylbutanoate	-	-	-	-	-	-	-	-	-	0.7	-
59	1575	1577	1,15-Hexadecadiene *	-	0.2	-	-	0.2	-	-	0.4	-	-	0.3
60	1577	1577	Citronellyl 3-methylbutanoate	-	-	-	-	-	-	-	-	-	0.8	-
61	1578	1579	*cis*-3-Hexadecene	0.2	0.3	-	-	0.2	-	-	0.3	-	-	-
62	1578	1580	*cis*-3-Hexenyl benzoate	-	-	-	-	-	0.1	-	-	-	-	0.5
63	1599	1600	Hexadecane	0.4	0.6	1.2	-	1.3	0.1	0.2	0.5	0.3	1.8	1.5
64	1654	1651	τ-Muurolol	-	-	-	0.6	-	-	-	-	-	-	-
65	1663	1665	2-Methylhexadecane	0.6	1.6	0.7	-	1.0	0.3	0.7	0.6	0.4	0.7	0.9
66	1669	1667	6,9-Heptadecadiene	9.0	15.5	0.8	-	3.2	1.2	1.1	12.4	6.4	1.4	6.1
67	1676	1676	*trans*-8-Heptadecene	3.8	4.9	0.4	0.3	1.1	0.6	0.4	4.0	2.0	0.7	1.9
68	1700	1700	Heptadecane	4.2	7.3	5.2	0.9	10.1	5.1	6.2	3.3	3.2	5.2	9.3
69	1713	1714	2-Pentadecanol	-	0.8	-	-	0.2	-	-	-	-	-	-
70	1770	1770	3-Methylheptadecane	0.1	0.8	0.2	-	0.7	0.4	0.4	-	-	0.3	0.6
71	1799	1800	Octadecane	0.3	0.3	1.4	-	0.7	0.3	0.5	0.3	1.1	0.7	0.7
72	1814	1812	2-Hexadecanol	-	0.2	-	-	-	-	-	-	-	-	-
73	1820	1819	Hexadecanal	-	-	-	-	-	0.1	-	-	-	-	-
74	1848	1845	Hexahydrofarnesyl acetone	-	0.3	0.2	-	0.6	0.4	0.5	-	0.5	0.6	0.7
75	1863	1864	2-Methyloctadecane	-	0.3	-	-	0.3	0.1	-	-	-	-	-
76	1873	1874	9-Nonadecene	0.4	0.7	0.5	-	0.3	1.2	0.2	1.7	0.3	0.4	0.8
77	1879	1879	1,18-Nonadecadiene *	-	1.4	-	-	-	-	-	-	-	-	-
78	1889	1892	1-Nonadecene	-	1.2	-	-	-	-	-	-	-	-	-
79	1900	1900	Nonadecane	4.5	5.4	5.2	1.6	12.4	7.4	13.3	5.2	3.8	1.5	12.6
80	1915	1909	2-Heptadecanol	-	1.2	-	-	0.5	-	0.2	-	-	-	-
81	1917	1920	Heptadecanal	0.2	-	-	-	-	-	-	0.2	-	-	-
82	1922	1919	Farnesyl acetone	-	-	-	-	-	0.3	-	0.2	0.3	-	-
83	1971	1972	3-Methylnonadecane	0.3	0.6	0.2	0.2	0.7	0.4	0.5	0.2	0.2	-	0.5
84	1992	1984	Hexadecanoic acid	-	-	-	-	0.3	-	-	0.2	0.3	-	-
85	1999	2000	Eicosane	0.2	0.2	0.4	0.3	0.4	0.3	0.5	0.3	0.8	0.2	0.2
86	2027	2024	Octadecanal	-	-	-	-	-	0.4	-	-	-	-	-
87	2066	2069	Linoleyl alcohol	-	0.4	-	-	-	-	-	-	-	-	-
88	2067	2064	2-Methyleicosane	0.2	-	-	0.2	0.2	-	-	0.2	0.2	-	-
89	2075	2080	1-Octadecanol	-	-	-	-	-	-	-	0.2	-	-	-
90	2100	2100	Heneicosane	3.9	2.1	1.9	4.6	3.9	2.2	5.5	3.9	4.3	0.8	3.3
91	2174	2172	3-Methylheneicosane	0.4	0.3	0.1	0.8	0.4	0.2	0.5	0.4	0.4	-	0.4
92	2199	2200	Docosane	0.9	0.5	1.0	1.3	0.7	0.4	1.3	0.7	1.5	0.7	0.5
93	2230	2229	Eicosanal	-	-	-	-	-	0.2	-	-	-	-	-
94	2262	2264	2-Methyldocosane	0.4	0.3	0.2	0.8	0.4	0.1	0.4	0.2	0.2	0.2	0.2
95	2272	2273	1-Eicosanol	-	-	0.1	0.2	-	0.1	-	0.2	-	-	-
96	2300	2300	Tricosane	9.4	9.1	8.6	13.1	12.3	4.8	14.3	7.5	10.8	8.6	11.7
97	2370	2372	3-Methyltricosane	1.1	1.6	0.9	2.0	1.8	0.4	1.9	0.8	0.8	0.9	1.4
98	2399	2400	Tetracosane	0.8	0.8	1.2	1.0	1.2	0.5	1.4	0.6	1.3	1.3	0.9
99	2459	2462	2-Methyltetracosane	0.2	0.4	0.2	0.4	0.4	0.1	0.3	0.1	0.3	0.3	0.2
100	2500	2500	Pentacosane	3.5	4.1	4.6	4.8	7.9	2.6	7.3	3.3	5.7	6.1	5.7
101	2570	2572	3-Methylpentacosane	0.6	1.6	0.8	0.9	2.2	0.4	1.4	0.5	0.8	1.3	1.1
102	2599	2600	Hexacosane	0.2	0.2	0.2	0.2	0.4	0.1	0.4	0.2	0.4	0.4	-
103	2659	2663	2-Methylhexacosane	-	0.2	-	-	-	-	-	-	0.3	-	-
104	2699	2700	Heptacosane	1.1	1.4	0.8	1.3	2.0	0.5	1.9	1.2	2.0	1.8	1.4
105	2770	2772	3-Methylheptacosane	0.4	0.9	0.2	0.4	0.7	-	0.5	0.4	0.8	0.5	0.4
			Sum	99.6	99.2	99.5	98.9	99.4	98.9	99.7	98.7	99.5	99.0	99.4

^a^ RI_det_, retention index determined relative to C7–C30 saturated alkanes run under identical chromatographic conditions; ^b^ RI_ref_, reference retention index retrieved from online databases of NIST Chemistry WebBook [18] (https://webbook.nist.gov/chemistry/, accessed on 20 March 2021) and ChemSpider [19] (https://www.chemspider.com/, accessed on 21 March 2021). Certain RI_ref_ values were retrieved from our previous studies [8,9]; ^c^ Compounds identified by mass spectra and retention indices; ^d^ Code for cultivar name, see Table 1. Percentages of individual components are presented as mean value of triplicate samples (RSD basically below 10% for major components); tr, trace, percentages lower than 0.05%; -, not detected; * Compounds tentatively identified.

**Table 3:** Ranking of the 20 essential oil compounds in the compound target network.

No. ^a^	Compound Name	Degree Value ^b^
33	Nerol	29
34	Citronellol	29
36	Geraniol	28
44	Geranic acid	28
4	*cis*-3-Hexen-1-ol	27
6	1-Hexanol	27
15	*trans*-β-Ocimene	21
35	Neral	21
47	Phloroglucinol trimethyl ether	21
17	*cis*-Linalool oxide furan	20
18	*trans*-Linalool oxide furan	20
26	Hydroquinone dimethyl ether	20
20	Nonanal	19
21	Linalool	19
37	Geranial	19
41	Citronellyl acetate	18
2	Hexanal	17
32	4,7-Dimethylbenzofuran	15
22	*cis*-rose oxide	13
24	Benzeneethanol	9

^a^ No., numbering of essential oil compounds, see Table 2; ^b^ Degree value of the compound in the compound target network as demonstrated in Figure 6.

**Table 4:** Ranking of all the 14 core targets in the PPI core network.

Target ^a^	Full Name of the Target	Degree Value ^b^
SLC6A4	Sodium-dependent serotonin transporter	11
CNR1	Cannabinoid receptor 1	10
ACHE	Acetylcholinesterase	10
HTR1A	5-Hydroxytryptamine receptor 1A	10
DRD2	Dopamine D2 receptor	10
MAOA	Monoamine oxidase type A	9
CHRNA4	Neuronal acetylcholine receptor subunit alpha-4	9
NR3C1	Glucocorticoid receptor	8
OPRM1	Mu-type opioid receptor	8
HTR2A	5-Hydroxytryptamine receptor 2A	7
MAOB	Monoamine oxidase type B	7
CHRM2	Muscarinic acetylcholine receptor M2	6
PTGS2	Prostaglandin G/H synthase 2	4
EGFR	Epidermal growth factor receptor	3

^a^ The standard gene name representative of each target; ^b^ Degree value of each target in the present core network (14 targets) of PPI as demonstrated in Figure 10c.

## Data Availability

The data are contained within this article.

## Supplementary Materials

The following supporting information can be downloaded at: https://www.mdpi.com/article/10.3390/molecules27092829/s1, Table S1: Results of drug-likeness screening for the 45 essential oil compounds; Table S2: The list of the 190 predictive potential targets for the 20 essential oil compounds; Table S3: Main topological parameters for the total network of protein—protein interactions.

## Sample Availability

Samples of the compounds are not available from the authors.
