# Supplementary material for: Chemical Diversity and Potential Target Network of Woody Peony Flower Essential Oil from Eleven Representative Cultivars (Paeonia × suffruticosa Andr.)
Source: Molecules. 2022 Apr 29;27(9):2829. doi: 10.3390/molecules27092829 (PMC9102020; doi:10.3390/molecules27092829)
Supplement: Supplementary file 1 [file molecules-27-02829-s001.zip › molecules-1689271-supplementary.pdf]

Supplementary Materials

Chemical diversity and potential target network of woody peony flower essential oil from eleven representative cultivars (*Paeonia* × *suffruticosa* Andr.)

Gaoming Lei *, Chaoying Song, Xinyue Wen, Guoyu Gao and Yanjie Qi

Department of Pharmaceutical Sciences, School of Basic Medical Sciences, Henan University of Science and Technology, 471023, Luoyang, China; 200320201100@stu.haust.edu.cn (C.S.); 191418030220@stu.haust.edu.cn (X.W.); 191418030201@stu.haust.edu.cn (G.G.); 171415020114@stu.haust.edu.cn (Y.Q.)

* Correspondence: leigm@haust.edu.cn

Legends

Table S1. Results of drug likeness screening for the 45 essential oil compounds

Table S2. The list of the 190 predictive potential targets for the 20 essential oil compounds

Table S3. Main topological parameters for the total network of protein – protein interactions

References

Table S1. Results of drug likeness screening for the 45 essential oil compounds [a]

No.[b]	Compound	Results on Lipinski's rule [c]	BBB permeant [d]
1	Octane	Lipinski: 1 violation: MLOGP>4.15	Yes
2	Hexanal	Lipinski: 0 violation	Yes
4	cis-3-Hexen-1-ol	Lipinski: 0 violation	Yes
6	1-Hexanol	Lipinski: 0 violation	Yes
15	trans-β-Ocimene	Lipinski: 0 violation	Yes
17	cis-Linalool oxide furan	Lipinski: 0 violation	Yes
18	trans-Linalool oxide furan	Lipinski: 0 violation	Yes
20	Nonanal	Lipinski: 0 violation	Yes
21	Linalool	Lipinski: 0 violation	Yes
22	cis-Rose oxide	Lipinski: 0 violation	Yes
24	Benzeneethanol	Lipinski: 0 violation	Yes
26	Hydroquinone dimethyl ether	Lipinski: 0 violation	Yes
32	4,7-Dimethylbenzofuran	Lipinski: 0 violation	Yes
33	Nerol	Lipinski: 0 violation	Yes
34	Citronellol	Lipinski: 0 violation	Yes
35	Neral	Lipinski: 0 violation	Yes
36	Geraniol	Lipinski: 0 violation	Yes
37	Geranial	Lipinski: 0 violation	Yes
41	Citronellyl acetate	Lipinski: 0 violation	Yes
44	Geranic acid	Lipinski: 0 violation	Yes
45	Tetradecane	Lipinski: 1 violation: MLOGP>4.15	No
47	Phloroglucinol trimethyl ether	Lipinski: 0 violation	Yes
49	2,6,10-Trimethyltridecane	Lipinski: 1 violation: MLOGP>4.15	No
51	Germacrene D	Lipinski: 1 violation: MLOGP>4.15	No
53	Pentadecane	Lipinski: 1 violation: MLOGP>4.15	No
54	α-Farnesene	Lipinski: 1 violation: MLOGP>4.15	No
63	Hexadecane	Lipinski: 1 violation: MLOGP>4.15	No
65	2-Methylhexadecane	Lipinski: 1 violation: MLOGP>4.15	No
66	6,9-Heptadecadiene	Lipinski: 1 violation: MLOGP>4.15	No
67	trans-8-Heptadecene	Lipinski: 1 violation: MLOGP>4.15	No
68	Heptadecane	Lipinski: 1 violation: MLOGP>4.15	No
71	Octadecane	Lipinski: 1 violation: MLOGP>4.15	No
76	9-Nonadecene	Lipinski: 1 violation: MLOGP>4.15	No
77	1,18-Nonadecadiene	Lipinski: 1 violation: MLOGP>4.15	No
78	1-Nonadecene	Lipinski: 1 violation: MLOGP>4.15	No
79	Nonadecane	Lipinski: 1 violation: MLOGP>4.15	No
80	2-Heptadecanol	Lipinski: 1 violation: MLOGP>4.15	No
90	Heneicosane	Lipinski: 1 violation: MLOGP>4.15	No
92	Docosane	Lipinski: 1 violation: MLOGP>4.15	No
96	Tricosane	Lipinski: 1 violation: MLOGP>4.15	No
97	3-Methyltricosane	Lipinski: 1 violation: MLOGP>4.15	No
98	Tetracosane	Lipinski: 1 violation: MLOGP>4.15	No

100	Pentacosane	Lipinski: 1 violation: MLOGP>4.15	No
101	3-Methylpentacosane	Lipinski: 1 violation: MLOGP>4.15	No
104	Heptacosane	Lipinski: 1 violation: MLOGP>4.15	No

[a] Essential oil components with percentages lower than 1.0% in all samples were excluded, which afforded 45 compounds; [b] Numbering of the essential oil compounds, see Table 2; [c] Drug likeness screening was performed on SwissADME using Lipinski's rule (http://www.swissadme.ch/, accessed on 2 April 2021) [1]; [d] Predictive results on blood brain barrier (BBB) permeation.

Table S2. The list of the 190 predictive potential targets for the 20 essential oil compounds [a]

Target [b]	Swiss-Prot ID [c]
ACHE	P22303
ADA	P00813
ADH1A	P07327
ADH1B	P00325
ADH1C	P00326
ADH7	P40394
ADRA1D	P25100
AKR1B1	P15121
ALDH1A1	P00352
ALDH1A3	P47895
ALOX5	P09917
AOC3	Q16853
APAF1	O14727
APOBEC3G	Q9HC16
AR	P10275
ATP12A	P54707
BCHE	P06276
BRD4	O60885
CA1	P00915
CA12	O43570
CA13	Q8N1Q1
CA14	Q9ULX7
CA2	P00918
CA4	P22748
CA5A	P35218
CA7	P43166
CA9	Q16790
CACNB1	Q02641
CACNG2	Q9Y698
CDC25A	P30304
CDC25B	P30305
CDK5R1	Q15078
CEL	P19835
CES1	P23141
CES2	O00748
CHRM1	P11229
CHRM2	P08172
CHRM3	P20309
CHRM4	P08173
CHRM5	P08912
CHRNA1	P02708

CHRNA3	P32297
CHRNA4	P43681
CHRNB2	P17787
CNR1	P21554
CNR2	P34972
CRHBP	P24387
CTRC	Q99895
CTSB	P07858
CTSK	P43235
CTSL	P07711
CYP11B1	P15538
CYP11B2	P19099
CYP17A1	P05093
CYP19A1	P11511
CYP2A6	P11509
CYP2C19	P33261
DAO	P14920
DHRS3	O75911
DHRS4	Q9BTZ2
DNM1	Q05193
DPP4	P27487
DPP7	Q9UHL4
DPP8	Q6V1X1
DRD2	P14416
DRD4	P21917
EGFR	P00533
ESR1	P03372
FAAH	O00519
FFAR1	O14842
FKBP1A	P62942
GABRA1	P14867
GABRA2	P47869
GABRD	O14764
GAMT	Q14353
GLI1	P08151
GLI2	P10070
GPBAR1	Q8TDU6
GRIA4	P48058
GRIN3A	Q8TCU5
GRM1	Q13255
GRM4	Q14833
GSK3B	P49841
GSR	P00390
HCAR2	Q8TDS4

HMGCR	P04035
HSD11B1	P28845
HSD17B2	P37059
HSD17B3	P37058
HTR1A	P08908
HTR2A	P28223
HTR2C	P28335
HTR3A	P46098
IDO1	P14902
IKBKB	O14920
JAK1	P23458
JAK2	O60674
KCNA1	Q09470
KCNA10	Q16322
KCNA2	P16389
KCNA3	P22001
KCNC3	Q14003
KCND1	Q9NSA2
KCNH2	Q12809
JMJD2C	Q9H3R0
LACTB	P83111
LRRK2	Q5S007
LTA4H	P09960
MAOA	P21397
MAOB	P27338
MAPKAPK2	P49137
MCL1	Q07820
MGLL	Q99685
MIF	P14174
MPI	P34949
MPO	P05164
MTNR1A	P48039
NCF1	P14598
NOS1	P29475
NOS2	P35228
NOTUM	Q6P988
NQO1	P15559
NQO2	P16083
NR0B1	P51843
NR1H4	Q96RI1
NR1I3	Q14994
NR3C1	P04150
NR3C2	P08235
OPRK1	P41145

OPRM1	P35372
PARP1	P09874
PDE10A	Q9Y233
PDE3A	Q14432
PDE4A	P27815
PDE4B	Q07343
PDE5A	O76074
PDE7A	Q13946
PDGFRA	P16234
PGR	P06401
PIK3CG	P48736
PNMT	P11086
PPARA	Q07869
PPARG	P37231
PPM1B	O75688
PRF1	P14222
PRKAB1	Q9Y478
PRKDC	P78527
PTAFR	P25105
PTGER2	P43116
PTGER3	P43115
PTGES	O14684
PTGS1	P23219
PTGS2	P35354
PTPN1	P18031
RARG	P13631
RARRES1	P49788
RBP1	P09455
RBP3	P10745
RDH11	Q8TC12
RDH12	Q96NR8
RDH13	Q8NBN7
RDH5	Q92781
RETSAT	Q6NUM9
RXRB	P28702
SAE1	Q9UBE0
SHH	Q15465
SIGMAR1	Q99720
SLC1A2	P43004
SLC6A2	P23975
SLC6A3	Q01959
SLC6A4	P31645
SLC8A1	P32418
SQLE	Q14534

SRD5A1	P18405
SRD5A2	P31213
STS	P08842
TAAR1	Q96RJ0
TAS2R31	P59538
TBXAS1	P24557
THRA	P10827
TPO	P07202
TRPA1	O75762
TRPM8	Q7Z2W7
VR1	Q8NER1
TRPV3	Q8NET8
TTR	P02766
TYMP	P19971
UGT2B17	O75795
UGT2B7	P16662
XDH	P47989

[a] The 20 essential oil compounds, see Table 3; [b] The standard gene name representative of each target; [c] The target ID according to UniProt (https://www.uniprot.org/, accessed on 28 May 2021) [2].

Table S3. Main topological parameters for the total network of protein – protein interactions [a]

Target	Degree	Closeness centrality	Betweenness centrality
DRD2	40	0.4764	6.585E-02
PTGS2	39	0.4946	0.1087
EGFR	36	0.4932	0.1172
HTR1A	34	0.4516	2.976E-02
CNR1	32	0.4561	3.562E-02
ESR1	31	0.4573	5.813E-02
CHRNA4	30	0.4344	2.855E-02
NR3C1	29	0.4815	7.292E-02
MAOA	29	0.4483	3.270E-02
OPRM1	29	0.4472	2.152E-02
AR	28	0.4417	4.227E-02
ACHE	26	0.4450	3.288E-02
OPRK1	26	0.4203	1.342E-02
MAOB	25	0.4303	2.267E-02
DRD4	25	0.4194	4.777E-03
SLC6A4	24	0.4407	1.867E-02
HTR3A	24	0.4213	2.218E-02
CHRM2	23	0.4354	2.374E-02
HTR2A	23	0.4344	1.856E-02
CYP19A1	22	0.4461	2.816E-02
ADRA1D	22	0.4072	1.416E-02
GRM1	22	0.3939	9.858E-03
PPARG	21	0.4655	4.443E-02
CNR2	21	0.4146	7.336E-03
SLC6A3	21	0.4136	1.775E-02
ALDH1A1	21	0.4081	4.246E-02
GRM4	21	0.4035	8.782E-03
CHRM1	20	0.3856	1.673E-02
PTGER3	19	0.4194	1.712E-02
UGT2B7	19	0.4108	1.900E-02
MPO	18	0.4213	2.992E-02
CYP17A1	18	0.3922	9.588E-03
HTR2C	18	0.3745	6.382E-03
CHRM4	17	0.3897	7.185E-03
UGT2B17	17	0.3848	1.358E-02
VR1	16	0.4396	1.841E-02
NOS2	16	0.4223	2.303E-02
ADH1B	16	0.4146	1.254E-02
GABRA1	16	0.3832	1.158E-02
CYP2C19	15	0.4203	2.260E-02
CHRNB2	15	0.3974	4.442E-03

SLC6A2	15	0.3939	2.544E-03
RBP1	15	0.3604	1.593E-02
JAK2	14	0.4242	2.028E-02
PGR	14	0.4184	6.220E-03
ALDH1A3	14	0.4090	1.820E-02
FAAH	14	0.4090	6.825E-03
HCAR2	14	0.3832	2.477E-03
GABRA2	14	0.3816	6.295E-03
HSD17B2	14	0.3784	1.654E-03
MTNR1A	14	0.3760	1.099E-02
CYP2A6	13	0.4213	1.692E-02
GSK3B	13	0.4018	5.702E-03
PTGS1	13	0.3991	6.280E-03
ADH7	13	0.3931	1.330E-02
ADH1A	13	0.3922	7.722E-03
TAS2R31	13	0.3753	0
MGLL	12	0.4127	1.293E-02
SLC1A2	12	0.4090	8.104E-03
HSD11B1	12	0.3792	8.326E-03
GRIA4	12	0.3760	4.955E-03
CYP11B2	12	0.3597	1.328E-03
SRD5A1	12	0.3583	9.870E-04
SRD5A2	12	0.3576	8.690E-04
HSD17B3	12	0.3576	8.550E-04
PARP1	11	0.4174	1.190E-02
PRKDC	11	0.3939	6.909E-03
MCL1	11	0.3914	6.829E-03
ALOX5	11	0.3914	4.897E-03
NR0B1	11	0.3792	3.062E-03
ADH1C	11	0.3745	3.790E-03
FFAR1	11	0.3493	7.808E-03
CYP11B1	11	0.3467	4.750E-04
KCNA1	11	0.3346	1.541E-02
NR1H4	10	0.3991	1.079E-02
GLI1	10	0.3948	6.835E-03
PTPN1	10	0.3914	3.312E-03
AKR1B1	10	0.3906	6.510E-03
NOS1	10	0.3897	5.260E-03
CHRNA3	10	0.3872	2.248E-03
BCHE	10	0.3832	7.582E-03
CES1	10	0.3832	5.877E-03
XDH	10	0.3800	8.545E-03
SHH	10	0.3684	1.210E-02
TRPA1	10	0.3655	1.640E-03

NR3C2	10	0.3618	1.384E-03
KCNA2	10	0.3576	1.366E-02
CHRM5	10	0.3480	4.630E-04
RDH12	10	0.3279	4.251E-03
GSR	9	0.3922	4.504E-03
PTGES	9	0.3776	2.922E-03
GRIN3A	9	0.3626	1.398E-03
PTAFR	9	0.3576	1.201E-03
RDH11	9	0.3487	3.934E-03
CHRM3	9	0.3370	6.400E-05
RDH5	9	0.3285	2.868E-03
LRRK2	8	0.3974	5.742E-03
PPARA	8	0.3881	3.849E-03
ADA	8	0.3760	1.837E-02
TTR	8	0.3662	1.644E-02
HMGCR	8	0.3626	1.410E-02
CDC25A	8	0.3590	1.326E-03
THRA	8	0.3576	3.490E-02
TRPM8	8	0.3548	1.058E-03
GABRD	8	0.3500	7.960E-04
RETSAT	8	0.3149	1.005E-03
RARG	7	0.3965	1.854E-02
PNMT	7	0.3864	3.893E-03
DAO	7	0.3655	3.619E-03
CES2	7	0.3590	1.003E-03
NR1I3	7	0.3555	2.149E-03
JAK1	7	0.3520	1.030E-03
DHRS4	7	0.3487	4.436E-03
CACNG2	7	0.3434	4.268E-03
KCNH2	7	0.3428	1.153E-02
DHRS3	7	0.3199	4.140E-04
DNM1	6	0.3906	1.987E-03
PDE4A	6	0.3881	4.113E-03
IDO1	6	0.3737	1.281E-02
ATP12A	6	0.3730	1.254E-02
GLI2	6	0.3611	3.330E-04
RXRB	6	0.3534	4.624E-03
DPP4	6	0.3514	1.090E-02
TBXAS1	6	0.3500	1.290E-04
TAAR1	6	0.3428	2.937E-03
CDC25B	6	0.3402	7.370E-04
GPBAR1	6	0.3377	2.965E-03
CHRNA1	6	0.3352	1.860E-04
SIGMAR1	6	0.3049	1.405E-02

PIK3CG	5	0.3808	4.672E-03
NQO1	5	0.3699	6.030E-04
CDK5R1	5	0.3677	2.950E-03
MAPKAPK2	5	0.3534	1.390E-03
CTSB	5	0.3480	1.258E-02
PTGER2	5	0.3473	2.753E-03
PDGFRA	5	0.3473	4.120E-04
STS	5	0.3440	4.003E-03
FKBP1A	5	0.3383	7.174E-03
LTA4H	5	0.3346	0
NCF1	5	0.3327	5.520E-04
DPP7	5	0.3262	6.046E-03
MIF	4	0.3730	5.697E-03
IKBKB	4	0.3507	8.580E-04
CRHBP	4	0.3487	4.970E-04
APAF1	4	0.3396	3.310E-05
CEL	4	0.3389	1.191E-02
PPM1B	4	0.3358	6.000E-04
PDE5A	4	0.3333	2.540E-04
CA2	4	0.3256	4.602E-03
SQLE	4	0.3256	3.590E-03
PDE4B	4	0.2984	4.900E-04
CACNB1	4	0.2950	6.990E-04
KCNA3	4	0.2839	2.600E-04
CA9	3	0.3669	0
TYMP	3	0.3583	1.386E-03
BRD4	3	0.3460	9.520E-05
AOC3	3	0.3339	0
TRPV3	3	0.3171	0
PRKAB1	3	0.3149	5.390E-04
SLC8A1	3	0.3013	1.642E-03
CTSK	3	0.2848	1.766E-03
RBP3	3	0.2673	0
CA7	2	0.3396	1.292E-03
CA4	2	0.3285	0
JMJD2C	2	0.3250	0
GAMT	2	0.3171	3.520E-04
MPI	2	0.3122	6.160E-04
RARRES1	2	0.2931	1.220E-04
DPP8	2	0.2696	0
CTSL	2	0.2642	5.840E-04
PDE10A	1	0.3233	0
RDH13	1	0.2903	0
NQO2	1	0.2737	0

APOBEC3G	1	0.2737	0
PRF1	1	0.2725	0
NOTUM	1	0.2696	0
TPO	1	0.2684	0
CA5A	1	0.2638	0
CA14	1	0.2638	0
CA13	1	0.2638	0
CTRC	1	0.2535	0
KCNA10	1	0.2510	0
KCNC3	1	0.2339	0

[a] The total PPI network for the 190 targets identified 183 connected nodes and 7 disconnected ones. The disconnected nodes were not included. The analysis was performed on STRING (https://cn.string-db.org/, accessed on 4 June 2021) [3]. Targets are represented by their standard gene names. The network was analyzed by Cytoscape software [4].

References

1. Daina, A.; Michielin, O.; Zoete, V. SwissADME: a free web tool to evaluate pharmacokinetics, drug-likeness and medicinal chemistry friendliness of small molecules. *Sci. Rep.* **2017**, *7*, 42717.
2. Consortium, T.U. UniProt: the universal protein knowledgebase in 2021. *Nucleic Acids Res.* **2020**, *49*, D480-D489.
3. Szklarczyk, D.; Gable, A.L.; Nastou, K.C.; Lyon, D.; Kirsch, R.; Pyysalo, S.; Doncheva, N.T.; Legeay, M.; Fang, T.; Bork, P.; et al. The STRING database in 2021: customizable protein–protein networks, and functional characterization of user-uploaded gene/measurement sets. *Nucleic Acids Res.* **2020**, *49*, D605-D612.
4. Shannon, P.; Markiel, A.; Ozier, O.; Baliga, N.S.; Wang, J.T.; Ramage, D.; Amin, N.; Schwikowski, B.; Ideker, T. Cytoscape: a software environment for integrated models of biomolecular interaction networks. *Genome Res.* **2003**, *13*, 2498-2504.
